# Risk Factors for Birth Asphyxia in an Urban Health Facility in Cameroon

**Published:** 2013

**Authors:** Andreas CHIABI, Seraphin NGUEFACK, Evelyne MAH, Sostenne NODEM, Lawrence MBUAGBAW, Elie MBONDA, Pierre-Fernand TCHOKOTEU, Anderson DOH FRCOG

**Affiliations:** 1 Yaounde Gynaeco-Obstetric and Pediatric Hospital,Faculty of Medicine and Biomedical Sciences, University of Yaounde I, Cameroon; 2 Université des Montagnes, Banganté, Cameroun; 3 Centre for the Development of Best Practices in Health, Yaounde Central Hospital, Avenue Henri Dunant, PO Box 87, Messa, Yaoundé, Cameroon.

**Keywords:** Birth asphyxia, Neonates, Hospital outcome, Cameroon

## Abstract

**Objective:**

The World Health Organization (WHO) estimates that 4 million children are born with asphyxia every year, of which 1 million die and an equal number survive with severe neurologic sequelae. The purpose of this study was to identify the risk factors of birth asphyxia and the hospital outcome of affected neonates.

**Materials & Methods:**

This study was a prospective case-control study on term neonates in a tertiary hospital in Yaounde, with an Apgar score of < 7 at the 5th minute as the case group, that were matched with neonates with an Apgar score of ≥ 7 at the 5th minute as control group. Statistical analysis of relevant variables of the mother and neonates was carried out to determine the significant risk factors.

**Results:**

The prevalence of neonatal asphyxia was 80.5 per 1000 live births. Statistically significant risk factors were the single matrimonial status, place of antenatal visits, malaria, pre-eclampsia/eclampsia, prolonged labor, arrest of labour, prolonged rupture of membranes, and non-cephalic presentation. Hospital mortality was 6.7%, that 12.2% of them had neurologic deficits and/or abnormal transfontanellar ultrasound/electroencephalogram on discharge, and 81.1% had a satisfactory outcome.

**Conclusion:**

The incidence of birth asphyxia in this study was 80.5% per1000 live birth with a mortality of 6.7%. Antepartum risk factors were: place of antenatal visit, malaria during pregnancy, and preeclampsia/eclampsia. Whereas prolonged labor, stationary labor, and term prolonged rupture of membranes were intrapartum risk faktors. Preventive measures during prenatal visits through informing and communicating with pregnant women should be reinforced.

## Introduction

The World Health Organization (WHO) describes birth asphyxia as failure to initiate and sustain default breathing at birth (1). WHO estimates that approximately 3% of about 120 million neonates born each year in developing countries develop asphyxia and need resuscitation. Approximately 900,000 of these newborns die as the result of asphyxia (1). Asphyxia accounts for 23% of neonatal deaths globally (2), and 8% of all deaths in children under five years of age (3). Reducing neonatal mortality is a critical component of achieving the fourth millennium development goal 4 (MDG 4) target of two-third reduction in deaths among children under 5 years of age (4).

In the short term, asphyxia could lead to multi organ dysfunction or even death (5,6), whereas in the long term, childhood survivors of neonatal hypoxic- ischemic encephalopathy might develop cerebral palsy; developmental delay; visual, hearing, and intellectual impairment; epilepsy; and learning and behavioral problems (5-9).

Unfortunately, in developing countries accurate epidemiological data is scarce, and the exact burden of severe neurological disability is unknown (10). The causes of birth asphyxia are heterogeneous and most occur before or during labour and delivery (11,12). Our objective was to determine the risk factors associated with neonatal asphyxia in order to develop preventive interventions in public health programs and thus to reduce neonatal mortality.

## Materials & Methods

We conducted a cross-sectional study on neonates born at the Yaounde Gynaeco-Obstetric and Pediatric Hospital (YGOPH) from May to September 2010. This hospital is a mother and child referral hospital in the capital city of Cameroon that provides services for the city of Yaounde and referrals from the rest of the country. Using a standard pre-tested questionnaire, data were collected from both mother and child at various time points. Maternal data were noted as medical history, age, gravidity, parity, number of antenatal care visits, gestational age, matrimonial status, multiple pregnancies, education, and profession. After delivery, neonates were evaluated and the Apgar score at the 5th minute was assessed, as well as the age, sex, and outcome at discharge from the hospital.

**Inclusion criteria**

We included all neonates with an Apgar scores < 7 at the 5th min. They were admitted in the neonatology unit, where they were followed up daily until discharge. A complete clinical assessment and a detailed neurological examination were done daily till discharge.

The control group was neonates with Apgar scores ≥ 7 at the 5th min, and each control was selected randomly after the recruitment of an asphyxiated newborn. They were equally followed up at the maternity until they and their mothers were discharged from the hospital.

Only Apgar scores were used to define asphyxia because we could not measure arterial umbilical cord pH in this hospital. We defined the severity of asphyxia in the same way as Amiel-Tieson and Ellison did (13), it was graded as moderate if Apgar score was £ 6, and severe if the score was £ 3.

For the case group, the Sarnat stage within the first 24 hours, type and duration of resuscitation, hospital outcome, and for both cases and controls the weight, sex duration of hospitalization were noted. This score permitted us to indicate the presence or not of hypoxic–ischemic encephalopathy.

Only the clinical elements of the Sarnat’s staging (14) were used, so that the electroencephalogram (EEG) was not systematically done in all the asphyxiated cases; Stage I lasted less than 24 hours and was characterized by hyperalertness, uninhibited Moro and stretch reflexes, sympathetic effects, and normal electrocardiogram; Stage II was characterized by obtundation, hypotonia, strong distal flexion, and multifocal seizures. The EEG showed a periodic pattern that was sometimes preceded by continuous delta activity; Stage III is maked by stupor, flaccidity and suppressed brainstem and autonomic functions. The EEG was isopotential or with infrequent periodic discharges.

All this information was recorded on a preconceived data entry form.

**Exclusion criteria**

Excluded from the study (cases and controls) were neonates who were delivered out of the YGOPH, neonates with one or multiple malformations incompatible with life, all premature babies, and all neonates whose parents refused to participate in the study.

**Sample size**

The study population was a consecutive sample over the study period and the sample size was determined using Kelsey formula (15):

$N=\frac{\{{\left.{Z_{\alpha }\sqrt[{2}]{2\mu (1-\mu )}+Z}_{\beta }\sqrt[{2}]{f\left(1-f\right)+p_{3}q_{3}}\right\}}^{2}}{({f-p_{3})}^{2}}$

μ=1/2f{1+R/[1+f(R-1)]}=0.25

a = level of significance = 0.05, Zα= 1.96 

β= power = 0.80 or 80%, Zβ= 0.84

R= odds ratio = 2

Q3 = 1-p3

According to Zupan et al.’s study (16), the amniotic fluid could be meconial in 50% of cases with asphyxia and in 20% of deliveries without asphyxia. Considering the cases (f) to be 0.5 and controls (p3) 0.2, the minimal size

was 96 (48 cases and 48 controls).

**Data analysis**

Data analysis was performed using SPSS software version 12.0 and Stata software version 8. The chi- squared test (significance level at p<0.05) and the odds ratio (OR) were used to analyze the data.

**Ethical considerations**

Authorization to conduct this study was obtained from the hospital authorities, and from the Ethical Committee of the hospital, and in the delivery room, an informed verbal consent to participate in the study was obtained

from all the mothers.

## Results

**A- Incidence**

From May 2010 to September 2010, 1117 deliveries were carried out at the YGOPH. Ninety were asphyxiated neonates (with an Apgar score < 7 at the 5th minute), giving an incidence of 80.5 per 1000. Also, we recorded 90 controls with an Apgar scores ≥ 7 at the 5th minute. Six cases (6.7%) had severe asphyxia (Apgar £ 3 at 5th minute) and, 84 (93.3%) moderate (Apgar between 4 and 6 at 5th minute).

**B- Sex**

Fifty (56 %) were boys and, 40 (44 %) girls, giving a sex ratio of 1.3

**C- Socio-demographic risk factors of the mothers**

Among the socio-demographic factors studied, only the difference in marital status was statistically significant (p=0.039), with 54.4% of mothers of asphyxiated neonates were unmarried, against 38.8% in the control group (Table 1).

**Table 1:** Demographic Risk Factors of The Mothers

		**Case**		**Control**		
		**N**	**(%)**	**N**	**(%)**	
**Mother’s age**	< 20 years	5	5.6	5	5.6	**p=0.541**
	20-34 years	71	78.9	73	81.1	
	>34 years	14	15.6	12	13.3	
**Profession**	Salaried	13	14.5	16	17.7	**p=** **0.435**
	Liberal	22	24.4	27	30	
	Students	19	21.1	22	24.5	
	Unemployed	36	40	25	27.8	
**Level of education**	Illiterates	1	1.1	1	1.3	**p=** **0.09**
	Primary	16	17.8	7	7.7	
	Secondary	56	62.2	60	66.6	
	University	17	18.9	22	24.4	
**Marital status**	Married	41	45.6	55	61.2	**p=** **0.039**
	Single	49	54.4	35	38.8	

**D- Antepartum risk factors**

Among the above antenatal variables studied, only the place of antenatal visits significantly influenced the occurrence of asphyxia. Most (46.7%) of the asphyxiated neonates had done their prenatal visits in primary health facilities, against 36.7% in the YGOPH and 15.6% in other hospitals.

Prenatal visits in primary health facilities significantly increased the occurrence of asphyxia (OR=3.81, CI 95%=1.8-7.7; p=0.01) (Table 2).

**Table 2:** Antepartum Risk Factors

		**Case**		**Control**		
		N	(%)	N	(%)	
**Parity**	Primiparous	29	32.2	26	28.8	**p=** **0.511**
	Multiparous	43	47.8	47	52.3	
	Grand	18	20.0	17	18.9	
**Gestational age**	37-42 weeks	79	87.7	83	92.3	**p=** **0.18**
	>42 weeks	11	12.3	7	7.7	
**Number of PNVs**	None	1	1.1	0	0.0	**p=** **0.61**
	1-3	7	7.8	7	7.7	
	>4	82	91.1	83	92.3	
**Place of PNVs**	YGOPH	33	36. 7	53	58.8	**p=** **0.01**
	Other hospitals	14	15.6	19	21.2	
	Health centers	42	46.7	18	20.0	

PNVs= prenatal visits, YGOPH=Yaounde Gynaeco-Obstetric and Pediatric Hospital

**E- Maternal pathologies**

Seven categories of pathologies during pregnancy were identified in the mothers of neonates with asphyxia, and of these, malaria and pre-eclampsia/eclampsia had statistically significant effects on the occurrence of asphyxia with p=0.015 and p=0.046, respectively (Table 3)

**F- Intrapartum risk factors**

Prolonged labor (33.3%), arrested labor (21.1%), prolonged membrane rupture (15.5%) non-vertex presentation (15.5%), and cesarean delivery (45.6%) significantly influenced the occurrence of asphyxia with p=0.000, 0.005, 0.007, 0.001, and 0.001, respectively (Table 4). The odds ratios were 2.03 (CI95% =0.45-6.6) for cesarean against 0.23 (CI95%=0.1-0.47) for vaginal delivery, and 1.34 (CI 95%=1.1-4.03) for the non-vertex presentation against 0.63 (CI95%=(0.19-2.01) for the vertex.

**G- Hypoxic –ischemic encephalopathy**

Forty-four (48.8%) out of the 90 asphyxiated neonates, developed hypoxic-ischemic encephalopathy. Most (51%) of them were in Sarnat’s stage I, 26% in stage II, and 23% in stage III.

**H- Hospital outcome**

Out of the 90 cases with asphyxia, 73 (81.1%) had a satisfactory outcome, 11 (12.2%) discharged with complications, and 6 (6.7%) died. Complications denoted an abnormal neurological examination and/or abnormal EEG or transfontanellar ultrasound (TFU). Half of the neonates with severe asphyxia (Apgar £ 3) died and the other half developed complications and the difference were statistically significant. (p=0,001). Deaths and complications were inversely correlated with the Apgar score (R= -0.22).

The mean duration of hospitalization was 6 days (range, 1-20 days) for the asphyxiated neonates, and 2.29 (range, 1-6 days) for normal babies.

All the neonates who died and 20.4% of those who developed complications were in Sarnat stage II.

There was a statistically significant correlation between Sarnat stage and the risk of death (p=0.001). The higher the Sarnat stage, the greater was the risk of dying (R=0.615).

**Table 3:** Maternal Pathologies

**Pathologies**	**Cases**		**Controls**		**P**
	**N**	**%**	**N**	**%**	
Malaria	20	22.2	14	15.6	0.015
Pre-eclampsia/ eclampsia	10	11.1	5	5.6	0.046
HIV	4	4.4	6	6.7	0.38
Diabetes	3	3.3	2	2.2	0.21
Bleeding in pregnancy	3	3.3	3	3.3	0.72
Hypertension	3	3.3	2	2.2	0.72
Uro-genital infections	1	1.1	2	2.2	0.73
No pathologies	46	51.11	56	62.2	0.64

**Table 4:** Intrapartum Risk Factors

**Factors**	**Cases**		**controls**		**P**
	**N**	**%**	**N**	**%**	
Prolonged labor	30	33,3	16	17.7	0.000
Arrest of labor	19	21,1	6	6.6	0.005
Prolonged rupture of membranes	14	15,5	4	4.4	0.007
Premature rupture of membranes	6	6,6	8	8.8	0.14
Cord prolapse	4	4,4	3	3.3	0.11
Presentation					0.001
Cephalic	72	80	85	94.5	
Breech	18	15.5	5	5.5	
Mode of delivery					0.001
Caesarean section	14	45.6	11	12.3	
Vaginally	49	54.4	79	87.7	
Instrumental delivery	3	3,3	1	1.1	0.57
Birth weight					0.16
< 2500 g	10	11.1	6	6.7	
2500-3999 g	70	77.8	78	86.6	
>4000 g	10	11.1	6	6.3	

## Discussion

**I- Epidemiology**

**I-1- Incidence**

The incidence of birth asphyxia in the present study was 80.5 per 1000, which was higher than 18.6 per 1000 obtained by Monebenimp et al. (17), at the Yaounde University Teaching Hospital in 1992, but lower than that 112 per 1000 obtained by Douba (18), at the Mother and Child Center of the Chantal Biya Foundation in Yaounde. Our hospital and the Mother and Child Center of the Chantal Biya Foundation are referral hospitals, where most complicated mother and child cases are referred. Compared to other African studies, this incidence is higher than 26.5 per 1000 reported by Airede et al. (19) in 1998, but lower than 100 per 1000 obtained by Ogunlesi et al. (20), both in Nigeria in 2003. In European studies, Thornberg et al. (21) in Sweden and Ganzales de Dios et al. (22) in Spain reported, respectively, incidences of 5.3 and 46 per 1000. This fluctuating incidence could be explained by the difference in the methodology used in different studies. The high incidence of asphyxia in the YGOPH is also because it is a referral centre, to which most primary health centers refer their cases with difficult or non-progressing deliveries.

**I-2- Sex**

Most of the neonates in our study (55.6%) were males, with a sex ratio of 1.3, although the difference between both sexes was not statistically significant. This predominance of the male sex was also reported by Douba in 2007 (18) at the Mother and Child Center of the Chantal Biya Foundation of Yaounde, Monebeninmp et al. at the Yaounde University Teaching Hospital in 1992 (17), and by Chandra et al. (23) in India in 1996, and also by Muhammad in Pakistan in 2004 (24), although no statistically significant relationship was established. On the other hand, Badawi et al. in Australia (12), found that the male sex increased the risk of occurrence of asphyxia by 50%, without any known cause-to-effect relationship. According to Johnston et al. (25), female sex hormones (estrogens) enhance the protection against anoxo-ischemic lesions.

**II- Socio-demographic risk factors of the mothers**

The only maternal socio-demographic risk factor which was found to be statistically significant, was the marital status. A possible explanation to this observation could be that the mothers living with their partners whether legally married or not, would better meet all their needs during pregnancy compared to single mothers, and the presence of the partners in the house provides social and financial support, and therefore, prompt regular antenatal consultations.

According to the study of Raatikainen et al., single status constitutes a risk factor for asphyxia and low birth weight during pregnancy (26). However, Houndjahoué in Mali (27), and Kinoti (28), in East Africa found that age less than 20 years, unemployment, and low level of education are other risk factors in addition to the marital status. On the other hand, Rehana et al. (29) in India noted that the risk of asphyxia increased with the mother’s age above 35 years, unemployment of the mother, or performing an intense physical activity, while Diallo et al. (30) in Guinea observed that a large proportion of asphyxiated neonates were born from uneducated mothers.

**III- Antepartum risk factors**

The major antepartum risk factors with statistical significance were: the site of antenatal consultation, malaria, and pre-eclampsia/eclampsia.

Houndjahouré in Mali (27), in addition to the above risk factors found in our study, noted grand multiparity, whereas other authors noted primarity in their respective studies (18,24,31). Concerning pathologies during pregnancy, Meka found malaria and uro-genital infections as major maternal pathologies in pregnancy (32), and Muhammad (24) reported bleeding in pregnancy, hypertension in pregnancy, eclampsia, and diabetes in the mother as major antepartum risk factors for asphyxia.

Prenatal visits in primary health facilities significantly influenced the occurrence of asphyxia. This could be explained by the fact that the women who go to health centers are poorly followed up due to lack of qualified staff, and referrals are done only in case of complications. Although most of the pregnant women (91.1%) had at least 4 antenatal consultations as recommended by WHO (33), this number is higher than that reported in the Demographic Health Survey in Cameroon in 2004, which stood at 83% (34). This goes to support the premise that what matters is not the number of consultations, but the quality of the care offered during the consultations. Malaria and preeclampsia/eclampsia were the main maternal risk factors identified. These findings have been noted by other authors (17,18,32,35).

Placental malaria, pre-eclampsia/eclampsia both lead to a decrease in placental blood flow, loss of placental integrity, and damage of endothelial cells. In placental malaria, in particular there can be an intervillous accumulation of inflammatory and infected red blood cells. All these phenomena can lead to an inadequate foeto-placental blood flow with foetal hypoxia causing growth retardation and birth asphyxia (36,37).

**IV- Intrapartum risk factors**

Prolonged labor, arrest of labor, prolonged rupture of membranes, cesarean section, and non-vertex presentation were the major statistically significant factors found in our study. These factors were also reported by other authors (17,23,24,29,35). We also found abnormal amniotic fluid (foul smell, meconium stained, yellowish) to be strongly associated with asphyxia, whereas Monebenimp et al. (17) only found a correlation between meconium-stained liquor and asphyxia. There was also a strong relationship between emergency caesarian section and neonatal asphyxia. This could be explained by the fact that most of the indications for the emergency cesarean sections were due to conditions which compromise adequate oxygen delivery to the foetus as prolonged labor, arrest of labor, hypertensive disorders in pregnancy, and cephalo-pelvic disproportions. Muhammad in 2004 in Pakistan (24), had similar findings. Chandra et al in India (23), found elective cesarean to be a risk factor for neonatal asphyxia and postulated that this might be due to some risk factors, which are not identified early in pregnancy, and which might cause acute foetal distress and consequently lead to asphyxia.

**V- Neonatal risk factors**

Amongst the foetal risk factors, only the non-cephalic presentation was statistically significant. Rehanna et al. (29) and Muhammad have reported similar findings (24). They also noted oligohydramnios and polyhydramnios to be major associated risk factors.

 **VI- Hypoxo-ischemic encephalopathy (HIE)**

Badawi et al. (12) in Australia observed a prevalence of encephalopathy of 3.8 per 1000 term live birth neonates, while Ellis et al. (38), in Nepal found a prevalence of 6.1 per 1000. Statistically significant antepartum risk factors found were: malaria and preeclampsia/eclampsia, and prolonged rupture of membranes were the main risk factors for developing HIE. Badawi et al. reported risk factors, such as socioeconomic status, family history of seizures or other neurological disease, conception after infertility treatment, maternal thyroid disease, severe preeclampsia, bleeding in pregnancy, viral illness, having an abnormal placenta, intrauterine growth restriction, and post maturity (12). Intrapartum risk factors were maternal pyrexia, persistent occipito-posterior position, acute intrapartum events, and operative vaginal delivery and emergency caesarean section (11). Ellis et al. in Nepal noted short maternal stature, high maternal age, primiparity, lack of antenatal care, and multiple births as independent preconceptual and antenatal risk factors for neonatal encephalopathy, and noncephalic presentation, prolonged rupture of membranes and the intrapartum complications of obstructed labour, cord prolapse, uterine rupture and oxytocin-induced labour as independent intrapartum risk factors (38).

**VII- Outcome**

We observed a favorable outcome in 81.1% of the neonates on discharge, whereas 12.2% had complications as abnormal neurological examination or abnormalities on the EEG and transfontanellar ultra sonogram. The mortality rate was 6.7%.

Douba (18), from a total of 356 cases with asphyxia had 4.2% deaths with 2.8% and 1.4% having severe and moderate neurological handicaps respectively. Monebenimp et al. (17) had a mortality rate of 5.4%, and Thornberg et al. (21) had 3% deaths and 2% complications.

**In conclusion,** this study demonstrates that perinatal asphyxia is a common clinical problem with a high morbidity and mortality rate and could lead to cerebral sequelae with a subsequent socio-economic burden on the families. It could be prevented to a large extent by informing and educating pregnant women on the follow-up of pregnancy and delivery, and appropriate management of pathological disorders during pregnancy and delivery. To achieve this, training and capacity building of health personnel in health facilities for timely diagnosis and referring of high risk pregnancies should be emphasized. Reinforcement of the technical capacity of the personnel of delivery wards should be done for adequate foetal monitoring during labor and delivery. These measures offer the best perspective for the prevention of birth asphyxia in this context.

## Notes

**Funding: **None.
